# Two new species of *Myriospora* (*Lecanoromycetes, Acarosporomycetidae, Acarosporales, Acarosporaceae*) from Europe, one last observed in 1925

**DOI:** 10.3897/mycokeys.139.200273

**Published:** 2026-08-25

**Authors:** Kerry Knudsen, Tereza Pušová, Jana Kocourková, Eva Kondrysová

**Affiliations:** 1 Department of Ecology, Faculty of Environmental Sciences, Czech University of Life Sciences Prague, Kamýcká 129, Praha - Suchdol, 165 00, Czech Republic Department of Ecology, Faculty of Environmental Sciences, Czech University of Life Sciences Prague Praha Czech Republic

**Keywords:** *
Acarospora
*, β-tubulin, iron-rich limestone, *Myriospora hassei*, *M. smaragdula*, extinct lichens, rare lichens, whewellite

## Abstract

We describe *Myriospora hilitzeri* from the Czech Republic, Germany and Italy. It is named after the Czech lichenologist and science fiction writer Alfred C. Hilitzer (1899–1940). The species *Acarospora smaragdula* form macrophylla was described by Magnusson from a single collection by A. C. Hilitzer from the summit of Keprník Mountain in Jeseníky Mountains of the Czech Republic on some gneiss rocks in 1925. It has never been collected again and is possibly extinct. It is raised to the species level in this paper as *Myriospora macrophylla*. A world-wide key of the 15 species of *Myriospora* is supplied.

## Introduction

*Myriospora* is a monophyletic genus in the *Acarosporaceae* (Crewe et al. 2006; Wedin et al. 2009; Westberg et al. 2011). The genus currently contains 15 species, including the two described in this study (Westberg et al. 2011; Arcadia and Knudsen 2012; Knudsen and Bungartz 2014; Knudsen et al. 2018, 2021; Purvis et al. 2018; Wang et al. 2025). So far, *Myriospora* species have been found on every continent except Australia and Africa. *Myriospora* is a monophyletic clade with all species having a tall hymenium, usually more than 150 µm, often thin paraphyses (approximately 1 µm wide), and interrupted algal layer (Crewe et al. 2006; Wedin et al. 2009). Secondary metabolites are not often found in this genus, but norstictic acid has been detected in *M. smaragdula* and *M. myochroa* (Westberg et al. 2011), and stictic acid was recently reported from the Chinese species *M. kunyuensis* (Wang et al. 2025). *Myriospora* occurs on a variety of HCl- rocks, with *M. smaragdula* occurring especially on copper-rich rock, and with *M. scabrida* also reported on soil, but no species has been reported on HCl+ calcareous rocks (Westberg et al. 2011).

In this paper, we describe a new species, *Myriospora hilitzeri* from Czech Republic, Germany, and Italy. *Myriospora smaragdula* form macrophylla, described from the Czech Republic, is recognized as a species because of its distinctive morphology (Magnusson 1929). It is possibly extinct.

## Material and methods

### Herbarium study

Specimens were studied from the herbaria PRM, TSB, and the private collections of Jana Kocourková and Kerry Knudsen (hb. K&K), Jiří Malíček (hb. Malíček), and Ulf Schiefelbein (hb. U.S.) Morphological features were examined using dissecting microscopes. Hand-cut sections were studied in water under compound microscopes at up to 1000× magnification. The amyloid reaction of the hymenial gel and subhymenium was tested in fresh, undiluted IKI (Merck’s Lugol solution; see protocol in Knudsen and Kocourková 2018, Knudsen et al. 2024). Ascus staining was examined in IKI following Hafellner (1993). Thin-layer chromatography (TLC) was performed in solvent systems A and C following the protocols of Orange et al. (2001) to check for secondary metabolites.

### Photographs

Macrophotographs were taken using an Olympus DP74 digital camera mounted on an Olympus SZX16 stereomicroscope. Images were stacked using the DeepFocus 3.5 module within the PROMICRA QuickPHOTO CAMERA 3.3 software. Microphotographs were taken with the same camera mounted on an Olympus BX51 compound microscope equipped with Nomarski interference contrast. Final figure plates were prepared using the Figure Maker module integrated in the same software.

### DNA extraction, amplification, sequencing, and phylogenetic analyses

DNA was extracted from 7 dried herbarium specimens using the Invisorb Spin Plant Mini Kit, following the manufacturer’s protocol with slight modifications: DNA was eluted in 50 μL instead of 100 μL, and samples were incubated in elution buffer for 15 minutes before the final centrifugation step. Extracted DNA was stored at –20 °C. DNA quality was verified on a 1% agarose gel, and concentration and purity were measured using a UVS-99 spectrophotometer (ACTGene). Four genetic markers were targeted: the nuclear ribosomal internal transcribed spacer (nrITS; White et al. 1990), the large subunit of the nuclear ribosomal DNA (nrLSU; Vilgalys and Hester 1990), the small subunit of the mitochondrial ribosomal DNA (mtSSU; Zoller et al. 1999), and the coding sequence of the β-tubulin gene (β-TUB; Einax and Voigt 2003). These loci were amplified by PCR using 1 μL of template DNA (20–25 ng), 10 μL of 2× MyTaq Red DNA Polymerase mix (Bioline), 8.2 μL of water, and 0.4 μM of each primer (10 μM), in a total reaction volume of 20 μL. PCR conditions for ITS and mtSSU included: initial denaturation at 95 °C for 5 min, followed by 5 cycles (95 °C for 33 s, 56 °C for 30 s, 72 °C for 30 s), 10 cycles (95 °C for 30 s, 54 °C for 30 s, 72 °C for 30 s), and 20 cycles (95 °C for 30 s, 50 °C for 30 s, 72 °C for 30 s), with a final extension at 72 °C for 15 min. For nrLSU, the program was: initial denaturation at 95 °C for 1 min, followed by 5 cycles (95 °C for 30 s, 55 °C for 30 s, 72 °C for 60 s) and 30 cycles (95 °C for 30 s, 52 °C for 30 s, 72 °C for 60 s), with a final extension at 72 °C for 10 min ; and for the β-tubulin: initial denaturation 95 °C for 1 min, followed by five cycles (95 °C for 30 s, 60 °C for 30 s, and 72 °C for 60 s) and finally 30 cycles (95 °C for 15 s, 55 °C for 30 s, and 72 °C for 45 s), with a final extension 72 °C for 10 min. PCR products were visualized on 1.0% agarose gels stained with ethidium bromide and purified enzymatically using ExoSap-IT™ Express Reagent (Thermo Fisher Scientific) following the manufacturer’s protocol. Purified PCR products, water, and forward primer (8 μL in total volume) were sequenced by BIOCEV, Vestec, CZE. Sequences were checked against the UNITE and NCBI databases for contamination. The sequences were proofread and concatenated manually into a single data set using SEQUENCHER® version 5.4.6. Sequences were aligned using the multiple sequence alignment online service MAFFT version 7.110 with ‘‘G-INS-I’’ strategy (Katoh and Toh 2008, http://mafft.cbrc.jp/alignment/server/). Indels longer than 1 bp were coded by the simple gap coding method (Simmons and Ochoterena 2000) as implemented in SEQSTATE 1.4.1 (Müller 2005). A partition homogeneity test (ILD) with heuristic search was performed under one thousand replicates by PAUP* version 4.0a169 (Swofford 2002) to determine whether the partitions were homogeneous. The PHT result indicated that incongruence was not significant (P value < 0.1). For phylogenetic study, the GTR+I+G4 model was selected as the best-fitting model of nucleotide substitution based on the Akaike Information Criterion using JMODELTEST 2.1.10 for each gene (Darriba et al. 2012). Phylogenetic trees were constructed using MRBAYES 3.2.2 (Ronquist and Huelsenbeck 2003). Input data was formatted for MRBAYES via the FABOX online converter tool to create a MRBAYES input file (http://birc.au.dk/~palle/php/fabox/fasta2mrbayes.php; Villesen 2007). *Pycnora sorophora* was used for the outgroup. A selection of representative species from five other genera recognized in the family, *Acarospora*, *Sarcogyne*, *Glypholecia*, *Timdalia*, and *Trimmatothelopsis*, support of the monophyletic clade of *Myriospora* in the family. For the *Myriospora* clade we continued the progression of phylogenies of *Myriospora* in Westberg et al. 2011, Purvis et al. 2018, Knudsen et al. 2021. Three replicate analyses with four chains each were computed 30,000,000 generations, sampling every 1000^th^ generation. After this number of runs, the average standard deviation of split frequencies reached a value lower than 0.01, indicating that convergence was reached. The data was also analyzed using maximum likelihood (ML). Three searches for ML analyses were executed under the GTR+GAMMA nucleotide substitution model, in RAxML v.8.2.10 (Stamatakis 2014). The substitution model was applied based on the evolutionary model calculation in RAxML. The Bayesian phylogenetic tree with posterior probability and bootstrap approximation with 1,000 replicates for ML method was visualized using FigTree v.1.4.0 and rooted with *Pycnora sorophora* (Rambaut 2012). The final alignment is accessible in the ZENODO database (https://zenodo.org/) under submission DOI https://doi.org/10.5281/zenodo.15812403. The final alignment contained 2894 concatenated characters, consisting of 1–495 (ITS), 496–1391 (mtSSU), 1392–2087 (nLSU), 2088–2894 (β-TUB). Of these characters, 1930 were constant, 168 were variable and parsimony-uninformative and 592 were parsimony informative.

## Results and discussion

**Figure 1.**
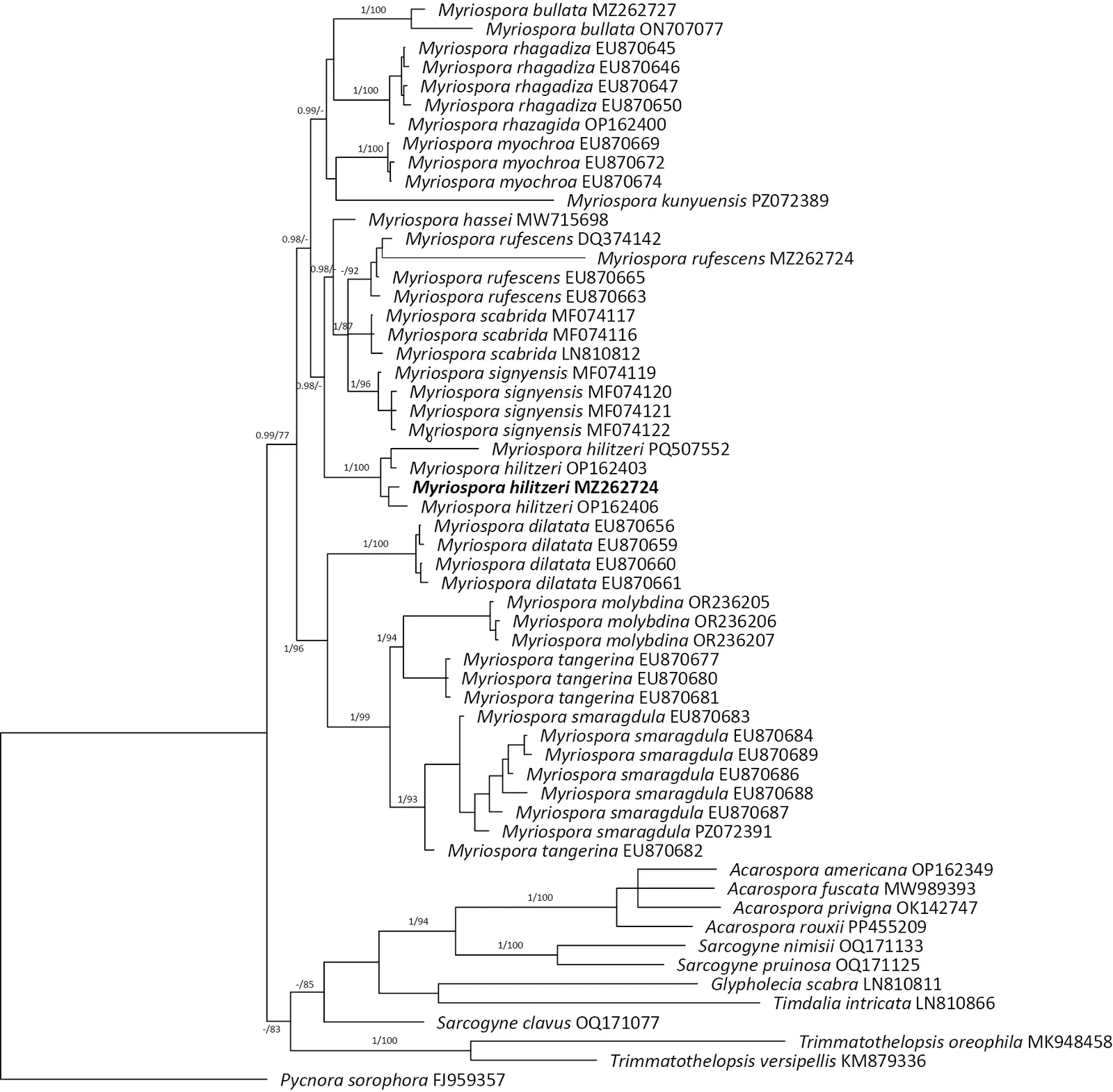
Bayesian inference tree obtained by phylogenetic analysis using a combined data set of ITS, mtSSU, nLSU, and β-TUB sequences of 57 members of *Acarosporaceae*. Specimen IDs (ITS GenBank numbers) in the tree correspond to species IDs with all GenBank numbers in Suppl. material 1. Bayesian posterior probability (BPP ≥ 0.95) and maximum likelihood bootstrap values (ML ≥ 70%) are indicated above branches (BPP/ML). *Pycnora sorophora* was used as an outgroup. The holotype of *Myriospora hilitzeri* is in bold.

The concatenated dataset (ITS, mtSSU, nrLSU and β-tubulin) resulted in a well-supported phylogeny of *Myriospora*, supporting the monophyly of the genus (Fig. 1). The phylogeny is congruent with previous *Myriospora* phylogenies, all which used the same specimens and added the new species sequences to each consecutive phylogeny (Westberg et al. 2011; Purvis et al. 2018; Knudsen et al. 2021). The four new sequences of *M. hilitzeri* form a well-supported clade separated from all other species in the genus except *M. macrophylla* and *M. westbergii*. *Myriospora macrophylla* was not included in the phylogenetic analysis due to the lack of material for DNA extraction. However, its unique morphology, especially the large squamules up to 10 mm wide and numerous apothecia, clearly distinguishes it from all other species of the genus. The absence of recent collections despite repeated searches at the type area suggests that the species may be extinct. *Myriospora westbergii* is known from single holotype from the Galapagos Islands in South America and has not been sequenced. *Myriospora westbergii* differs from *M. hilitzeri* in having a thalline margin (Knudsen and Bungartz 2014).

In our phylogeny, we also included a specimen of *Myriospora smaragdula* PZ072391 from Canada. It was recovered within the *M. smaragdula* clade (Fig. 1). The specimen was collected on iron-rich calcareous rock, a substrate not previously recorded for this species.

The addition of *Myriospora hilitzeri* and *M. macrophylla* brings the total number of accepted species in *Myriospora* to fifteen. We do not accept *Myriospora himalayensis* as a member of the genus though it was published as a *Myriospora* (Mishra et al. 2021). The species was described as a *Myriospora* on basis of a high hymenium, small ascospores, norstictic acid and pruinose apothecia. None of these characters are restricted to *Myriospora*. Interrupted algal layers were not mentioned in the protologue. A picture of the holotype shows some discontinuous narrow hyphal bands penetrating an algal layer, but this can be found in the description of many *Acarospora* species with *A. praeruptorum* being just one example (Knudsen et al. 2026). The holotype and the only specimen needs a phylogenetic analysis. We do not doubt it is a valid species but that it is a regional lineage of the *Acarospora* or *Sarcogyne* clade with a high hymenium.

## Taxonomy

### Myriospora hilitzeri

Taxon classificationFungiProtococcidiidaGrelliidae

K. Knudsen, Pušová, & Kocourk. sp. nov.

FAC44D37-D745-5201-B47E-DC2B72F5CD84

862526

Fig. 2

#### Type.

Czech Republic • North Moravia: E Sudetes, Jeseníky Mts, Studénková hole, Červená hora, W-facing slope above the spring ‘Vřesová studánka’, alt. 1322 m, 50.14583, 17.13622, overhanging schist rock, 26 April 2019, Z. Palice 27581 (holotype PRA, isotype PRM).

#### Diagnosis.

Similar to *Myriospora myochroa* but differs in having smooth to lumpy areoles vs. distinctly convex areoles, 1–4 apothecia, up to 10 when in process of replicating into several areoles vs. 1–4(–14) apothecia in single undivided areole/squamule, having a narrower parathecium indistinct to 15–30 µm wide vs. up to 90 µm wide, and a lower hymenium 150–170 µm high vs. up to 230 µm high, and in not producing norstictic acid.

#### Etymology.

Named in honor of the Czech lichenologist Alfred C. Hilitzer (1899–1940), who was the collector of this species in 1920s. He was also a science fiction novelist publishing in 1933, Mitigen, gas against war, in which a scientist creates a gas that makes people not want to go to war.

#### Description.

Thallus of areoles (1.0–)1.5–2.0(–4.0) mm wide, but mostly 2 mm wide, 0.5–0.6 mm thick, irregular in shape, rarely convex, broadly attached and slightly elevated on mycelial base, usually beginning to replicate by division when ca. 1.0 mm wide, sometimes forming clusters up to 4 mm with up ca. 10 apothecia and in process of dividing into three to five areoles. Upper surface pale brown, smooth to lumpy. Lower surface white to brown. Epicortex indistinct to 10 µm thick, often absent. Cortex 45–70 µm thick, with mostly round cells, 3–5 µm wide. Algal layer usually 100–110 µm thick, partially interrupted by narrow bands 30–40 µm wide, algal cells not dense, 5–7 µm wide. Medulla up to 350 µm thick, sometimes obscure, intricate hyphae, 2–3 µm wide, sometimes disarticulated. Apothecia (0.1–)0.2–0.5 mm wide, immersed or slightly elevated, disc reddish brown to almost black. Parathecium indistinct to 15–30 µm wide, merging with the cortex sometimes forming a ring around immersed apothecia, or narrow and having the color of the thallus to almost black around elevated apothecia. Hymenium 130–170 µm, paraphyses 1 µm wide, with apices unexpanded in narrow gelcap up to 2 µm wide, epihymenium 15–30 µm tall, light brown, hymenal gel IKI+ red or in squash light blue turning to red (hemiamyloid). Asci 70–80 × 7–13 µm, ascospores 2–3(–4) × 1–3 µm, variable from thin to broadly ellipsoid (n = 50).

**Figure 2.**
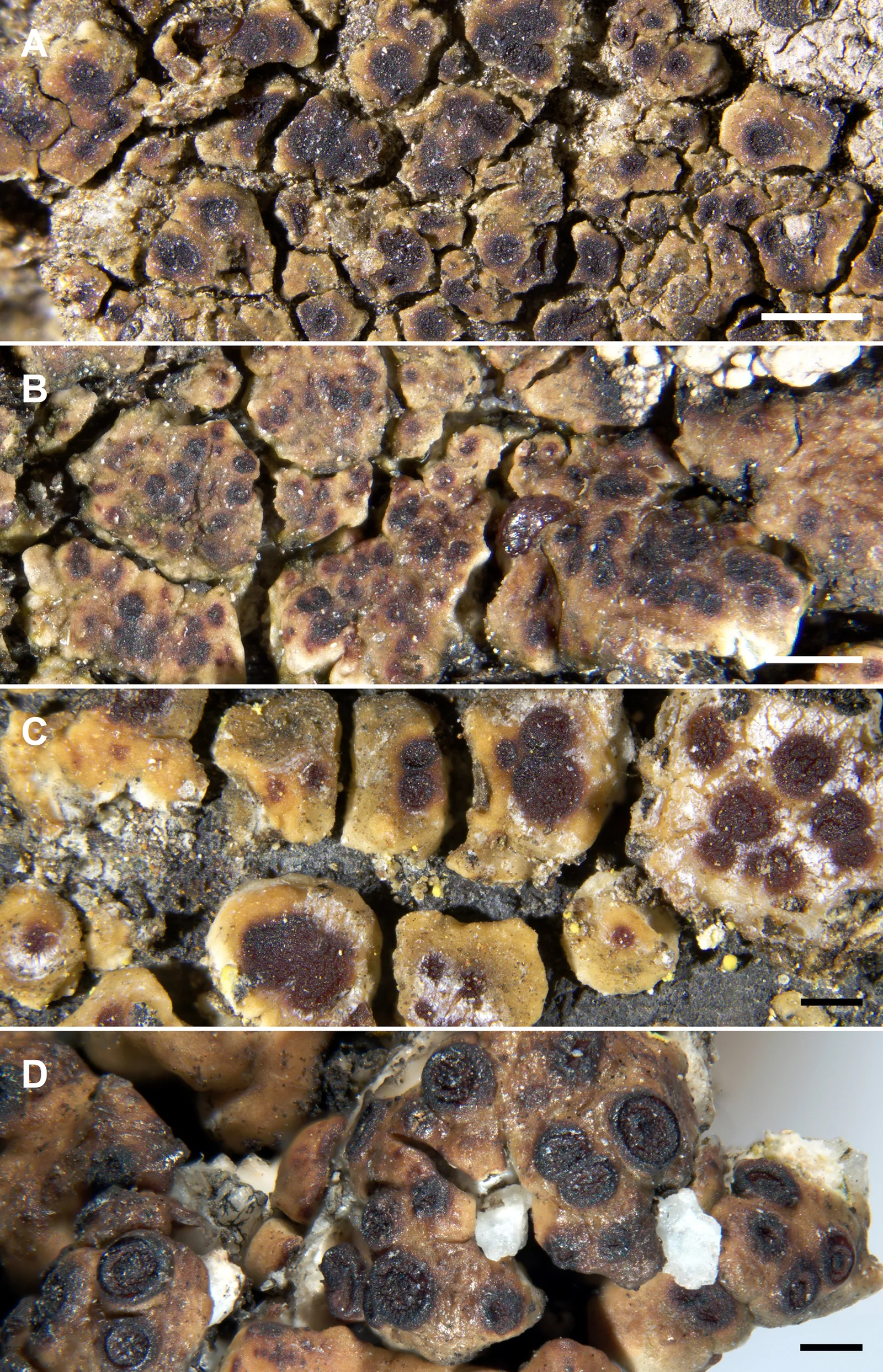
*Myriospora hilitzeri*. Palice 27581, holotype (**A**), Malíček 8554 (**B**), Malíček et al. 1303 (**C**), Bouda, PRM 953588 (**D**). **A**. Habit of the areolate thallus with apothecia; **B**. Thallus areoles on process of replicating areoles with up to 10 young apothecia; **C**. Areoles with apothecia without conspicuous parathecium; **D**. Convex areoles with apothecia showing distinct parathecium. Scale bars: 1 mm (**A, B**); 500 µm (**C, D**).

Subhymenium 50–60 µm tall, with oil drops, IKI+ blue. Hypothecium indistinct, up to 20 µm thick. Pycnidia 40 µm tall × 95 µm wide, conidiogenous cells 10–14 × 2 µm, conidia globose or ellipsoid 2(–3) × 1 µm (n = 10). Not producing secondary metabolites.

#### Distribution and ecology.

Czech Republic, Germany, Italy on siliceous rock, sometimes in underhangs, at elevations from 130 to 1420 m in Central Europe and Italy at 1750 m. Claude Roux may have reported *Myriospora hilitzeri* as *M. hassei* from France (Roux et coll. 2025). His reports of *M. hassei* are not based on sequenced material.

#### Additional specimens examined.

Czech Republic, illegible, A. Hilitzer, 4. Sept. 1921 (PRM 693531); • Šumava: Jezerní stěna, ad rupium latera impendentiva, 6. Aug.1926, A. Hilitzer, det. A. H. Magnusson as *Acarospora lesdainii* (PRM 693527 & 693528); • Central Bohemia, distr. Příbram, Vltava River valley, Nalžovice, Drbákov–Albertovy skály National Nature Reserve, slopes of Albert’s rocks, 350–450 m, siliceous rock, 18. Apr. 2008, J. Malíček 1303 et al. (hb. Malíček); • Bartůňkova vyhlídka, NW-facing rocky ridge, 49.72172, 14.36669, alt. 340 m, Fe-rich volcanic rock, 12. Nov. 2021, J. Malíček 14788 (hb. Malíček); • Northern Bohemia, distr. Liberec, Jizerské hory Mts. (protected landscape area) Polední kameny, alt. 1006 m, N 50.85597, 15.21810, on rock, 16.9.2010, F. Bouda (PRM 860080); • Jizerské hory Protected Landscape Area, Bílý Potok, Jizerskohorské bučiny National Nature Reserve, Frýdlantské cimbuří rocks, 50.85592, 15.21776, alt. 910 m, granite rock, J. Malíček & J. Steinová, 10. June 2021, Malíček 14928 (hb. Malíček); • Krkonoše, Čertova zahrádka, infected, 13. July 1924 A. Hilitzer (PRM 693530); • Silesia, distr. Jeseník, Hrubý Jeseník Mts, Bělá pod Pradědem, Šerák-Keprník National Nature Reserve, rocks on top of Mt Keprník (1423 m), 50.17083, 17.11639, alt. 1420 m, in underhang of siliceous rock, 26. Apr. 2019, J. Malíček 12893 & dupl., F. Bouda & L. Syrovátková (hb. Malíček); • Jeseníky PLA, Bělá pod Pradědem, Kamenné okno geological structure on ridge N of Mt Červená hora (1333 m), 50.14922, 17.13647, alt. 1300 m, siliceous rock, 18. Aug. 2015, J. Malíček 8554, V. Lenzová, J. Starosta & L. Zemanová (hb. Malíček); • distr. Bruntál, Hrubý Jeseník Mts., Karlova Studánka, Ovčárna, Petrovy kameny, top of Mt Petrovy kameny, alt. 1440 m, 50.06855, 17.23388, on acidic phylite rock, 20 Aug. 2015, J. Kocourková 11477 (hb. K&K); • Germany, Sachsen-Anhalt, Harz, Harz National Park, Mittlere Zeterklippe (Central Zeter rock), alt. c. 860 m, 51.80694, 10.64111, exposed rocks, granite, 1 June 2018, U. Schiefelbein 4789, (hb. Schiefelbein); • c. 2.5 km NE of Schierke, unnamed cliff between Leistenklippe and Bärenklippe, alt. c. 870 m, 51.77929, 10.69502, exposed granite rocks, 10 Sept. 2016, U. Schiefelbein 4472, (hb. Schiefelbein); • ~1.5 km N of Schierke, Ahrensklimt (top), alt. c. 800 m, 51.77321, 10.66695, exposed granite rocks, with *Endococcus stigma*, 1 June 2018, U. Schiefelbein 4478 & 4480 (hb. Schiefelbein); • Saalkreis, Friedrichsschwerz, dry grassland E of the village, alt. c. 130 m, 51.55000, 11.86028, boulders in grassland, 7 Nov. 2019, U. Schiefelbein 5712 (hb. Schiefelbein); • Italy, Piemonte, Prov. Cuneo: Alpi Marittime, Rocca dell’Abisso W of Colle di Tenda, just N below the summit, 44.14306, 7.50417, ca. 2750 m, cliffs of gneiss, 22. July 2000, M. Tretiach with J. Hafellner (TSB 34105); • Calabria, Prov. Reggio Aspromonte Pietra Impiccata, 1750 m, in niche (overhanging rocks) of schistose rocks, Aug. 1988, P. L. Nimis, Tretiach & Castello (TSB 11545).

#### Notes.

The Italian specimens of *M. hilitzeri* were determined as *M. myochroa* without norstictic acid (Knudsen et al. 2018). Some Czech specimens were also misdetermined as *M. myochroa* (Malíček et al. 2026). *Myriospora hilitzeri* has usually smooth areoles or squamules with 1–4 immersed apothecia (more when in process of replicating by division), a parathecium indistinct to 15–30 µm wide and does not produce norstictic acid. *Myriospora myochroa* differs in having convex areoles or squamules with 1–4(–14) immersed apothecia, in having a parathecium up to 90 µm wide, and in producing norstictic acid (Westberg et al. 2011). Czech specimens determined as *M. myochroa* need to be re-examined. The species may not occur in Czech Republic.

Magnusson determined a collection of *Myriospora hilitzeri* by Hilitzer as *A. smaragdula* var. lesdainii. But var. lesdainii is a synonym of *A. smaragdula* producing norstictic acid (Westberg et al. 2011).

Specimens of *Myriospora hilitzeri* from Germany and Czech Republic were also misidentified as *Myriospora scabrida* at an early stage of development (Knudsen et al. 2021). *Myriospora hilitzeri* differs from *M. scabrida* in sometimes having an elevated parathecial margin not wider than ca. 30 µm vs. a parathecial margin expanded up to 120 µm around elevated apothecia (Westberg et al. 2011).

Some specimens of *Myriospora hilitzeri* were determined by collectors as *M. tangerina* mainly based on the apothecia. *Myriospora hilitzeri* differs from *M. tangerina* (Westberg et al. 2011) in having a pale brown vs. tangerine orange thallus, in having a narrower parathecium to 30 µm wide vs. a parathecium expanding to 80 µm, in usually having a wider apothecial disc (0.1–) 0.2–0.5 (–0.8) mm vs. 0.1–0.2 mm diam, in having a lower hymenium 130–170 µm vs. 180–250 µm (Westberg et al. 2011).

**Figure 3.**
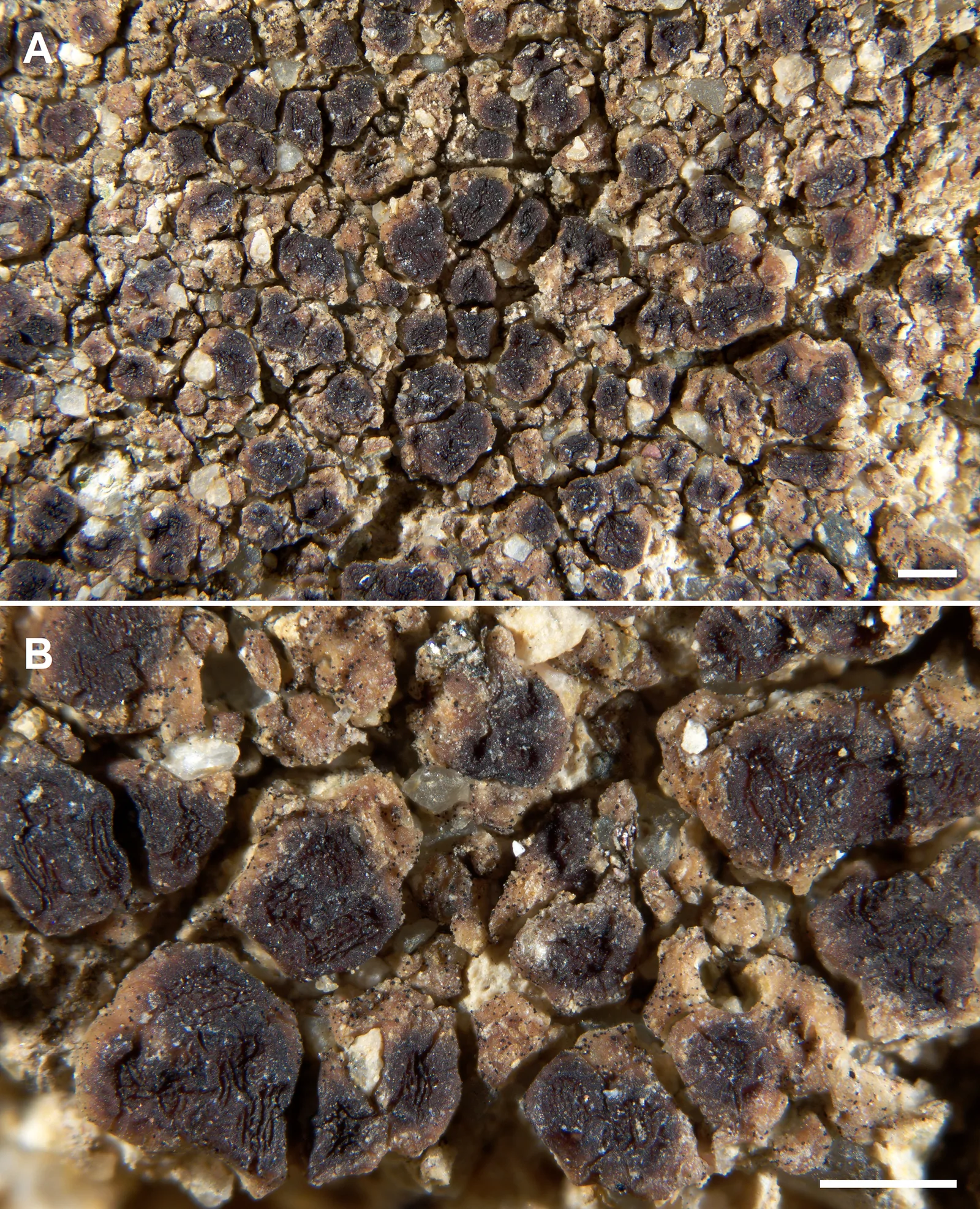
*Myriospora hassei*, Knudsen 707 (hb. K&K). **A**. Habit of the areolate thallus with apothecia; **B**. Detail of areoles with one to four apothecia. Scale bars: 1 mm (**A, B**).

*Myriospora hilitzeri* is somewhat similar to *M. hassei* with a ring around apothecia or slightly elevated apothecia formed from a parathecium indistinct to 30 µm wide (Fig. 3) but they are distinct species based on their sequences and phylogenetic positions (Fig. 1). *Myriospora hassei* is endemic to coast of California and Oregon and adjacent coastal mountains and is a facultative lichenicolous lichen on the yellow coastal species *Acarospora socialis* (Knudsen 2007; Knudsen and Bungartz 2014). Determinations of *M. hassei* from the Holarctic flora of North America as well as high elevations in the interior of United States need to be revised (Consortium of Lichen Herbaria 2025). Though some of these specimens were determined by the first author (K.K.), he considers these specimens as probably misdetermined and representing an undescribed taxon and could even be *M. hilitzeri* in the Holarctic flora of North America.

For our phylogeny we sequenced a specimen of *Myriospora smaragdula* PZ072391 from Canada collected by Jesuit lichenologist John McCarthy on calcareous rock rich in iron. *Myriospora smaragdula*, like other *Myriospora*, has never been reported on calcareous rock (Westberg et al. 2011). Although it is known to often grow on copper-rich rocks, *M. smaragdula* was never collected on iron-rich substrates (Westberg et al. 2011). The Canadian specimen was recovered in *M. smaragdula* clade in our phylogeny. Viktória Krajanová, a lichen curator at the Slovakia National Museum, examined the specimen with Raman spectroscopy looking for possible new mineral oxalates, but only found crystals of calcium oxalate monohydrate (the mineral whewellite), which is one of the most common oxalates found in lichens. The crystals were on the underside of squamules where they were in contact with the rock.

### Myriospora macrophylla

Taxon classificationFungiProtococcidiidaGrelliidae

(H. Magn.) K. Knudsen, Pušová, & Kocourk. comb. et stat. nov.

36B542D0-B453-5B08-BF49-3558F56D4A13

862527

Fig. 4

≡Acarospora smaragdula f. macrophylla H. Magn., K. svenska Vetensk-Akad. Handl., Ser. III 7(no. 4): 143 (1929), basionym.
≡Acarospora smaragdula subsp. macrophylla (H. Magn.) Clauzade & Cl. Roux, Bull. Mus. Hist. Nat. Marseille 41: 66 (1981).

#### Type.

Czech Republic, Silesia, distr. Jeseník, Hrubý Jeseník Mts, Bělá pod Pradědem, Šerák-Keprník National Nature Reserve, rocks on top of Mt Keprník, 50.17083, 17.11639, 1424 m, 5. Aug. 1925, Hilitzer s.n. (UPS, holotype, image viewed online at W3NEXDB (access with hyperlink), PRM 693545, isotype!).

#### Description.

Due to the rarity of the species and the possibility it is extinct this description is based on a description by Magnusson (1929), a short discussion by Hilitzer (1927), an examination with dissecting microscope without dissection of a small isotype annotated by Magnusson, TLC of small fragment, and at website of National Herbarium of the Czech Republic W3NEXDB the study of two pictures of the holotype at UPS.

**Figure 4.**
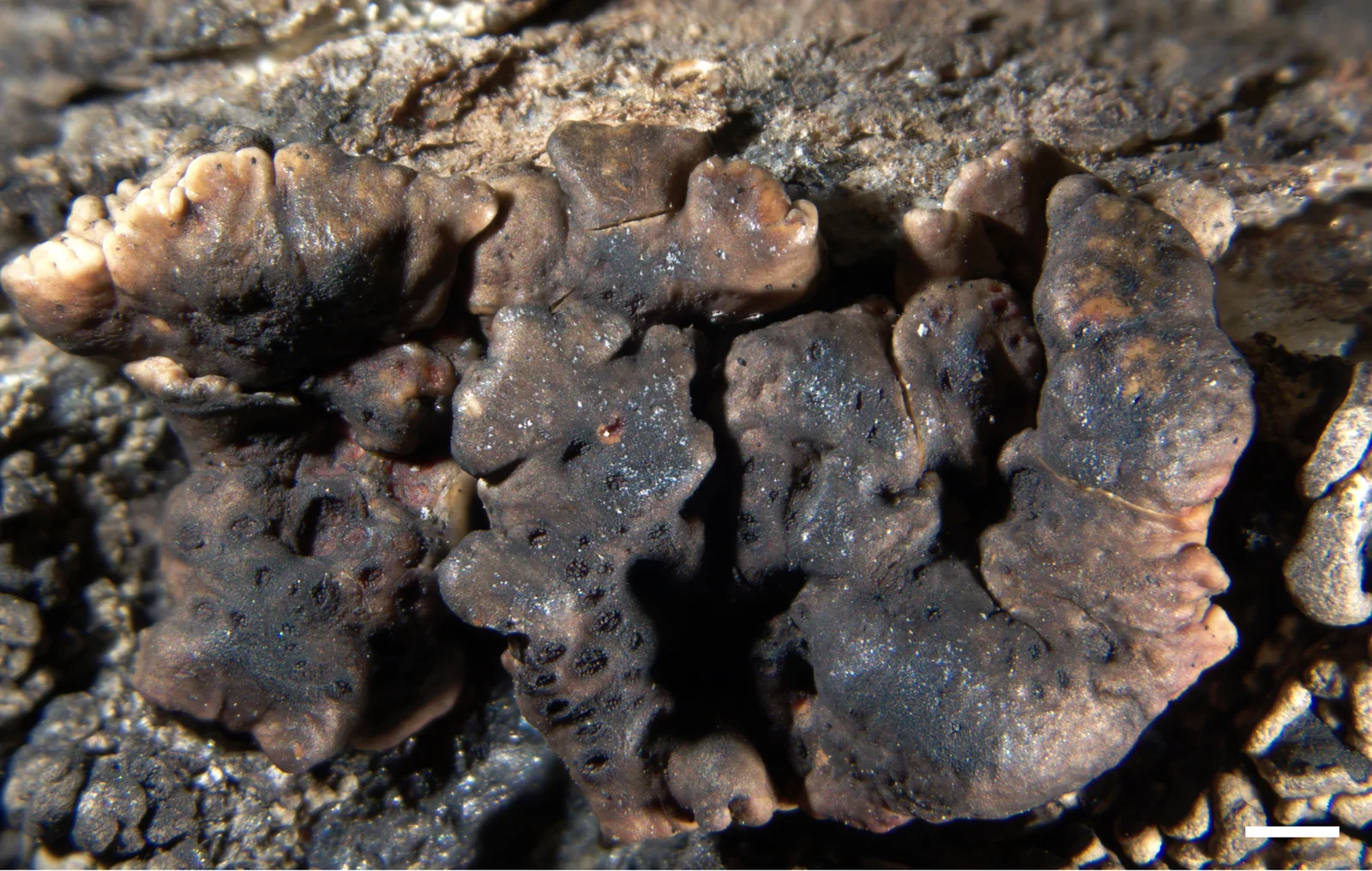
*Myriospora macrophylla*, Hilitzer s.n. (PRM 693545). Habit of the thallus with large squamules and numerous apothecia. Scale bar: 1 mm.

Squamules, 4–10 mm wide, up to 1.35 mm thick, on stout stipe, overshadowing adjacent lichens and destroying them and possibly saprobically absorbing them, upper surface pale to dark brown, very uneven with large swellings or flattened pustules, upper cortex 60–85 µm thick, an interrupted alga layer 200–300 µm thick, and medulla 500–700 µm thick, hyphae continuous with stipe. Apothecia 10–20, immersed, disc. 0.3–0.4 mm across convex, slightly darker than the thallus, ± blackish brown, with distinct parathecium, hymenium 130–170 µm high, thin paraphyses not capitate, asci narrow with the small ascospores, 3–4 × 1.4–1.7 µm, subhymenium very distinct. No pycnidia observed. Not producing secondary metabolites.

#### Ecology and distribution.

Czech Republic Silesia, Jeseníky Mountains, on summit Keprník Mountain, probably in full sun, 1424 m, on gneiss, known only from type collection. “At the summit of the Keprník, there are only a small number of low gneiss rocks. It was there that I found this species” (Hilitzer 1927).

#### Notes.

This species is only known from the holotype in UPS and the isotype in PRM. Magnusson originally annotated Hilitzer’s 1925 collection as *Acarospora murina* (Sandst.) var. macrophylla in ed. which was also written on the holotype and isotype packets (Hilitzer 1927). By 1929 Magnusson considered *A. murina* a form of *A. smaragdula* and published *A. smaragdula* f. macrophylla. *Myriospora macrophylla* differs from *M. smaragdula* not only in morphology with its robust squamules, but it also does not produce norstictic acid.

Jiří Malíček, F. Bouda and L. Syrovátková visited the summit of Keprník Mountain on April 26, 2019, but found only a specimen of *M. hilitzeri* in an underhang of rock. *Myriospora macrophylla* differs from *A. hilitzeri* in forming large squamules up to 10 mm wide and with up to 20 apothecia and was apparently growing fully exposed to sun and not in an underhang like *M. hilitzeri*.

Neither A. Vězda or any other Czech lichenologist from Suza and Servít to contemporary Czech lichenologists have rediscovered *Myriospora macrophylla*. Hilitzer himself never collected it again. There are no reports from other parts of Europe that we could find. It has conspicuous and large squamules which cannot be easily over-looked by any skilled lichenologist. We consider *M. macrophylla* to probably be extinct in the Czech Republic.

##### World key to *Myriospora*

**Table:** 

1	Thallus usually effigurate, with long narrow lobes (Holarctic)	***Myriospora molybdina* (Westberg et al. 2024)**
–	Not effigurate with long narrow lobes	**2**
2	Producing secondary metabolites	**3**
–	Not producing secondary metabolites	**5**
3	Producing stictic acid (China)	***M. kunyuensis* (Wang et al. 2025)**
–	Producing norstictic acid	**4**
4	Areoles, yellow to green, K+ red crystals, not K- and not needing TLC to detect norstictic acid (Holarctic, rare in South America)	***M. smaragdula* (Westberg et al. 2011)**
–	Areoles or squamules, whitish to gray brown or blackish brown, K+ red crystals or K- and needing TLC to detect norstictic acid (Europe)	***M. myochroa* (Westberg et al. 2011)**
5	With distinct black elevated parathecial ring 25–40 µm wide around immersed apothecia (Europe)	***M. rufescens* (Knudsen et al. 2021, see also Westberg et al. 2011)**
–	Without distinct black elevated parathecial ring around immersed apothecia	**6**
6	On seashores in salt spray zone and inland within a maritime influence on rock and walls of buildings (Atlantic Ocean, North America and Europe)	***M. rhagadiza* (Westberg et al. 2011)**
–	Not on seashores in salt spray or in maritime zone	**7**
7	Always red or orange, often with cortical or epicortical crystals	**8**
–	Not orange or red, without cortical or epicortical crystals	**10**
8	Distinctly red, often with cortical or epicortical crystals golden red, granular, in a 5–15 µm thick layer (Europe)	***M. dilatata* (Westberg et al. 2011)**
–	Distinctly orange	**9**
9	Distinctly orange, bullate, apothecia mostly punctiform, c. 0·1–0·2 mm diam, not bullate (Europe)	***M. tangerina* (Westberg et al. 2011)**
–	Usually orange (rarely brown without epicortical crystals), bullate, apothecia up to 0.5 mm wide (Europe)	***M. bullata* (Knudsen et al. 2021)**
10	Large squamules, 4–10 mm wide overshadowing other lichen (Czech Republic)	***M. macrophylla* ( this paper)**
–	Not large squamules overshadowing other lichens, 4–10 mm wide	**11**
11	Elevated lecideine apothecia, parathecium 50–120 µm wide	**12**
–	Elevated or immersed apothecia with thalline or narrower parathecial margin	**13**
12	Apothecia 1–7(–12) per areole/squamule, thallus with 10–20 μm thick epicortex (Southern Hemisphere, Antarctica ecoregion)	***M. signyensis* (Purvis et al. 2018)**
–	Apothecia 1–2(–4) per areole/squamule, thallus lacking epicortex (Northern Hemisphere, Asia, Greenland, Iceland, Europe)	***M. scabrida* (Westberg et al. 2011)**
13	Apothecia only in elevated thalline margins, parathecium barely distinct (South America)	***M. westbergii* (Knudsen and Bungartz 2014)**
–	Immersed and/or elevated apothecia often with ring or elevated margin color of thallus around apothecia, parathecium indistinct to 30 µm wide, merging with cortex	**14**
14	Europe (Czech Republic, Germany, Italy)	***M. hilitzeri* (this paper)**
–	North America (Pacific coast, California and Oregon, United States)	***M. hassei* (as *Acarospora hassei* , Knudsen 2007)**

## Supplementary Material

XML Treatment for Myriospora hilitzeri

XML Treatment for Myriospora macrophylla

## Additional information

### Artificial Intelligence (AI) use

The authors accept full responsibility for the content of the manuscript, including the disclosure of any use of AI.

No AI tools were used in the preparation of this manuscript.

### Author ORCIDs

K. Knudsen https://orcid.org/0000-0001-5419-5729

T. Pušová https://orcid.org/0009-0001-2566-5266

J. Kocourková https://orcid.org/0000-0001-5511-9752

E. Kondrysová https://orcid.org/0000-0002-8337-3253

### Data availability

All of the data that support the findings of this study are available in the main text or Supplementary Information.

## Supplementary materials

Supplementary material 1 Sampled specimens included in the molecular phylogeny Data type PDF https://binary.pensoft.net/file/1754236This dataset is made available under the Open Database License (http://opendatacommons.org/licenses/odbl/1.0/). The Open Database License (ODbL) is a license agreement intended to allow users to freely share, modify, and use this Dataset while maintaining this same freedom for others, provided that the original source and author(s) are credited. Kerry Knudsen, Tereza Pušová, Jana Kocourková, Eva Kondrysová

## References

[B1] Arcadia L, Knudsen K (2012) The name *Myriospora* is available for the *Acarospora smaragdula* group. Opuscula Philolichenum 11: 19–25. 10.5962/p.382080

[B2] Consortium of Lichen Herbaria (2025) Consortium of Lichen Herbaria. https://lichenportal.org/portal/index.php [Accessed July 2025]

[B3] Crewe AT, Purvis OW, Wedin M (2006) Molecular phylogeny of *Acarosporaceae (Ascomycota)* with focus on the proposed genus *Polysporinopsis*. Mycological Research 110: 521–526. 10.1016/j.mycres.2006.01.010 16616841

[B4] Darriba D, Taboada GL, Doallo R, Posada D (2012) jModelTest 2: More models, new heuristics and parallel computing. Nature Methods 9(8): e772. 10.1038/nmeth.2109 PMC4594756 22847109

[B5] Einax E, Voigt K (2003) Oligonucleotide primers for the universal amplification of β-tubulin genes facilitate phylogenetic analyses in the regnum Fungi. Organisms, Diversity & Evolution 3: 185–194. 10.1078/1439-6092-00069

[B6] Hafellner J (1993) *Acarospora* und *Pleopsidium* – zwei lichenisierte Ascomycetengattungen (*Lecanorales*) mit zahlreichen Konvergenzen. Nova Hedwigia 56: 281–305.

[B7] Hilitzer A (1927) Notes sur quelques lichens récoltés dans les Jeseníky. Preslia 5: 33–35.

[B8] Katoh K, Toh H (2008) Recent developments in the MAFFT multiple sequence alignment program. Briefings in Bioinformatics 9(4): 286–298. 10.1093/bib/bbn013 18372315

[B9] Knudsen K (2007) *Acarospora*. In: Nash TH III, Gries C, Bungartz F (Eds) Lichen Flora of the Greater Sonoran Desert Region. Volume 3. Lichens Unlimited, Arizona State University, Tempe, 1–38.

[B10] Knudsen K, Bungartz F (2014) *Myriospora westbergii* (*Acarosporaceae*), a new discovery from the Galapagos Islands, Ecuador. Opuscula Philolichenum 13: 177–183. 10.5962/p.386074

[B11] Knudsen K, Kocourková J (2018) Two new calciphytes from Western North America, *Acarospora brucei* and *Acarospora erratica* (*Acarosporaceae*). Opuscula Philolichenum 17: 342–350. 10.5962/p.386180

[B12] Knudsen K, Kocourková J, Schiefelbein U (2018) New reports of *Myriospora (Acarosporaceae)* from Europe. Mycotaxon 132: 857–865. 10.5248/132.857

[B13] Knudsen K, Kocourková J, Hodková E, Schiefelbein U (2021) A new species of *Myriospora (Acarosporaceae)* and a report of *Myriospora rufescens* from Central Europe. Herzogia 34: 327–338. 10.13158/heia.34.2.2021.327

[B14] Knudsen K, Kocourková J, Pušová T, Kondrysová E, Malíček J (2026) A study of *Acarospora nitrophila*, a rare and misunderstood lichen species, and the sister species *A. praeruptorum* (*Acarosporales*, *Lecanoromycetes*). Folia Cryptogamica Estonica, Fasc. 63: 25–36 10.12697/fce.2026.63.03

[B15] Knudsen K, Kocourková J, Peske O, Beck A, Velmala S, Wirth V (2024) Three poorly known *Acarosporaceae* of Central Europe: New reports from Czech Republic, Germany, and Italy. Herzogia 37(1): 5–15. 10.13158/heia.37.1.2024.5

[B16] Magnusson AH (1929) A monograph of the genus *Acarospora*. Kungliga Svenska Vetenskapsakademiens Handlingar, ser. 3: 7: 1–400.

[B17] Malíček J, Palice Z, Bouda F, Knudsen K, Šoun J, Vondrák J, Novotný P (2026) Atlas Českých Lišejníků. https://dalib.cz/en/data/info

[B18] Mishra GK, Nayaka AS, Upreti DK (2021) *Myriospora himalayensis* (*Acarosporaceae*, lichenized fungi), a new species from Western Himalayan region of India. Taiwania 6: 89–92.

[B19] Müller K (2005) SeqState: Primer design and sequence statistics for phylogenetic DNA datasets. Applied Bioinformatics 4: 65–69. 10.2165/00822942-200504010-00008 16000015

[B20] Orange A, James PW, White FL (2001) Microchemical Methods for the Identification of Lichens. British Lichen Society, London, 101 pp.

[B21] Purvis OW, Fernández-Brime S, Westberg M, Wedin M (2018) *Myriospora*, a genus newly reported for Antarctica with a worldwide key to the species. The Lichenologist 50: 101–112. 10.1017/S0024282917000652

[B22] Rambaut A (2012) FigTree, version 1.4.4, Institute of Evolutionary Biology, University of Edinburgh. http://tree.bio.ed.ac.uk/software/figtree

[B23] Ronquist F, Huelsenbeck JP (2003) MrBayes 3: Bayesian phylogenetic inference under mixed models. Bioinformatics 19(12): 1572–1574. 10.1093/bioinformatics/btg180 12912839

[B24] Roux et coll. (2025) Catalogue des lichens et champignons lichénicoles de France métropolitaine Tome 1. – Edit. Claude Roux, Mirabeau (Vaucluse).

[B25] Simmons MP, Ochoterena H (2000) Gaps as characters in sequence-based phylogenetic analyses. Systematic Biology 49(2): 369–381. 10.1093/sysbio/49.2.369 12118412

[B26] Stamatakis A (2014) RAxML version 8: a tool for phylogenetic analysis and post Analysis of large phylogenies, Version 4, Sinauer USA associates. 10.1093/bioinformatics/btu033 PMC3998144 24451623

[B27] Swofford DL (2002) PAUP*: Phylogenetic Analysis Using Parsimony (*and Other Methods), Version 4. Sinauer Associates, Sunderland. 10.1002/0471650129.dob0522

[B28] Vilgalys R, Hester M (1990) Rapid genetic identification and mapping of enzymatically amplified ribosomal DNA from several Cryptococcus species. Journal of Bacteriology 172(8): 4238–4246. 10.1128/jb.172.8.4238-4246.1990 PMC213247 2376561

[B29] Villesen P (2007) FaBox: an online toolbox for fasta sequences. Molecular Ecology Notes 7: 965–968. 10.1111/j.1471-8286.2007.01821.x

[B30] Wang JX, Liang FH, Zhou SN, Hu L (2025) *Myriospora kunyuensis* (*Acarosporaceae*, lichenized *Ascomycota*), a new species from the eastern coast of China. Phytotaxa 708(1): 68–76. 10.11646/phytotaxa.708.1.6

[B31] Wedin M, Westberg M, Crewe AT, Tehler A, Purvis OW (2009) Species delimitation and evolution of metal bioaccumulation in the lichenized *Acarospora smaragdula* (*Ascomycota*, Fungi) complex. Cladistics 25: 161–172. 10.1111/j.1096-0031.2009.00240.x 34879601

[B32] Westberg M, Crewe AT, Purvis OW, Wedin M (2011) *Silobia*, a new genus for the *Acarospora smaragdula* complex (*Ascomycota*, *Acarosporales*) and a revision of the group in Sweden. The Lichenologist 43: 7–25. 10.1017/S0024282910000617

[B33] Westberg M, Wedin M, Svensson M (2024) *Myriospora molybdina* comb. nov. and the identity of *Acarospora hysgina*. Nordic Journal of Botany 2024: e04269. 10.1111/njb.04269

[B34] White TJ, Bruns T, Lee S, Taylor J (1990) Amplification and direct sequencing of fungal ribosomal RNA genes for phylogenetics. In: Innis MA, Gelfand DH, Sninsky JJ, White TJ (Eds) PCR Protocols: A Guide to Methods and Applications. Academic Press, San Diego, 315–322. 10.1016/b978-0-12-372180-8.50042-1

[B35] Zoller S, Scheidegger C, Sperisen C (1999) PCR primers for the amplification of mitochondrial small subunit ribosomal DNA of lichen-forming *Ascomycetes*. The Lichenologist 31: 511–516. 10.1006/lich.1999.0220

