# Supplementary material for: Two new species of Myriospora (Lecanoromycetes, Acarosporomycetidae, Acarosporales, Acarosporaceae) from Europe, one last observed in 1925
Source: MycoKeys. 2026 Aug 25;139:73–87. doi: 10.3897/mycokeys.139.200273 (PMC13535497; doi:10.3897/mycokeys.139.200273)
Supplement: Supplementary file 1 [file mycokeys-139-073-s001.pdf]

Supl. Table 1 A list of sampled specimens included in the molecular phylogeny.

**Species**	**Origin**	**Voucher**	**nrITS**	**mtSSU**	**nrLSU**	**βtubulin**
Acarospora americana	U.S.A., Shell Creek, Road	Dart 1526.1 (hb. K&K)	OP162349	OP177737	OP216668	-
Acarospora fuscata	West Bohemia, Tachov, Sedmohoří	Kocourková 8499 & Knudsen (PRM)	MW989393	MW989442	-	-
Acarospora privigna	Germany	Kison S.M. (13/1) (hb.K&K)	OK142747	OK032132	ON303622	PZ221047
Acarospora rouxii	U.S.A., Arizona	Nash 35136 (ASU)	PP455209	PP455222	PP455234	
Glypholecia scabra	Norway, Oppland	Westberg 08-232 (S)	LN810811	LN810936	-	LN810703
Myriospora bullata	Germany, Sachsen-Anhalt	U. Schiefelbein 4763 (hb. U.S.)	MZ262727	MZ262739	MZ262739	PZ196604
Myriospora bullata	Germany, Sachsen-Anhalt	U. Schiefelbein 5323 (hb. U.S.)	ON707077	ON715673	ON725168	PZ221048
Myriospora dilatata	Sweden, Torne Lappmark	Baloch SW116 (S)	EU870656	EU870708	LN810872	EU870766
Myriospora dilatata	Sweden, Västerbotten	Crewe 75 (UPS)	EU870659	EU870711	-	EU870769
Myriospora dilatata	Sweden, Jämtland	Tibell 23546 (UPS)	EU870661	EU870713	-	EU870771
Myriospora dilatata	Sweden, Lycksele Lappmark	Nordin 5507 (UPS)	EU870660	EU870712	LN810871	EU870770
Myriospora hassei	U.S.A, California, Los Angeles Co.	Knudsen 707 (hb. K&K)	MW715698	MW715737	MW715726	PZ191629
Myriospora hilitzeri	CZE, Hruby Jesenik, Bělá pod Pradědem	Malíček 12893 (hb. Malíček)	OP162403	OP177788	OP216718	PZ170820
Myriospora hilitzeri	CZE, Central Bohemia, Sedlčany	Malíček 14788 (hb. Malíček)	PQ507552	-	PZ208921	PZ191626
Myriospora hilitzeri	CZE, Northern Bohemia, Bílý potok	Malíček 14928 ((hb. Malíček)	OP162406	OP177791	OP216721	PZ191627
Myriospora hilitzeri	CZE, Jeseníky, Studénkova hole	Palice 27587 (PRM)	MZ262724	MZ262736	MZ262746	PZ191628
Myriospora kunyuensis	China, Shandong Province, Kunyu Mountain	Wang SDNU 20242087 (PRM)	PZ072389	-	PZ208922	PZ196603
Myriospora molybdina	Norway, Finnmark	Westberg VAR092 (S F278293)	OR236205	OR236217	-	OR285162
Myriospora molybdina	Norway, Finnmark	Westberg VAR046 (S F278198)	OR236207	OR236219	-	OR285163
Myriospora molybdina	Norway, Finnmark	Westberg VAR080 (S F278291)	OR236206	OR236218	-	-
Myriospora myochroa	Finland, Lapponia Inarensis	Westberg LIN-159 (LD)	EU870669	EU870721	LN810874	EU870780
Myriospora myochroa	Sweden, Bohuslän, Strömsholm	Tibell 22675 (UPS)	EU870672	EU870724	-	EU870783

Myriospora myochroa	Sweden, Bohuslän, Havssten	Westberg & Westberg 06-120 (LD)	EU870674	EU870726	MF095737	EU870785
Myriospora rhagadiza	Sweden, Bohuslän	Westberg 06-040 (LD)	EU870647	EU870699	LN810875	EU870757
Myriospora rhagadiza	UK, Wales	Crewe A86 (UPS)	EU870650	EU870702	-	EU870760
Myriospora rhagadiza	Sweden, Bohuslän	Westberg & Westberg 06-121d (LD)	EU870645	EU870697	MF095738	EU870755
Myriospora rhagadiza	Sweden, Bohuslän	Westberg 06-034 (LD)	EU870646	EU870698	LN810876	EU870756
Myriospora rhazagida	Ziegelstein, Schleswig-Holstein	Dolnik 4239 (hb. Dolnik)	OP162400	OP177785	OP216716	PZ196605
Myriospora rufescens	UK, Wales, Parys Mt	Crewe A25 (UPS)	EU870665	EU870717	-	EU870776
Myriospora rufescens	CZE, Western Bohemia, Františkovy Lázně	Malíček 1783 (hb. Malíček)	MZ262716	MZ262728	-	-
Myriospora rufescens	Romania, Zlatna	Rusu (BM)	DQ374142	DQ374121	-	EU870773
Myriospora rufescens	UK, Wales, Parys Mt	Crewe 304 (UPS)	EU870663	EU870715	-	EU870774
Myriospora scabrida	Sweden, Härjedalen	Westberg 07-010 (LD)	MF074116	MF074130	-	MF074130
Myriospora scabrida	Sweden, Härjedalen	Westberg 07-009g (LD)	MF074117	MF074123	-	MF074129
Myriospora scabrida	Sweden, Härjedalen	Santesson 33077a (UPS L-529787)	LN810812	LN810937	-	LN810704
Myriospora signyensis	Antarctica, Signy Island	Smith RILS8673 (AAS)	MF074122	MF074127	-	MF074134
Myriospora signyensis	Antarctica, Signy Island	Purvis & Maltman OWPS2_8 (S)	MF074120	MF074126	-	MF074132
Myriospora signyensis	Antarctica, Signy Island	Smith RILS8668 (AAS)	MF074121	MF074128	-	MF074133
Myriospora signyensis	Antarctica, Signy Island	Purvis OWPS6_8 (BM)	MF074119	MF074125	-	MF074131
Myriospora smaragdula	Greenland, Tugtugtooq	Crewe 104(UPS)	EU870683	EU870735	-	EU870795
Myriospora smaragdula	Sweden, Härjedalen	Wedin 6620 (UPS)	EU870688	EU870740	LN810879	EU870800
Myriospora smaragdula	Sweden, Hälsingland	Ågren 384 (UPS)	EU870686	EU870738	LN810878	EU870798
Myriospora smaragdula	Norway, Troms	030807 Lättman (UPS)	EU870684	EU870736	-	EU870796
Myriospora smaragdula	UK, Cornwall	Wedin 7310 (UPS)	EU870689	EU870741	-	EU870802
Myriospora smaragdula	Sweden, Härjedalen	Wedin 6618 (UPS)	EU870687	EU870739	-	EU870799
Myriospora smaragdula	Canada, Labrador	McCarthy 4953(NFM)	PZ072391	-	PZ209147	PZ221046

Myriospora tangerina	Sweden, Jämtland	Tibell 23548 (UPS)	EU870681	EU870733	-	EU870793
Myriospora tangerina	Bohemia, Krkonose Mtns,	Halda & Palice 1382 (hb. Palice)	EU870677	EU870729	-	EU870788
Myriospora tangerina	Sweden, Härjedalen	Santesson 30943 (UPS)	EU870680	EU870732	-	EU870792
Myriospora tangerina	Sweden, Lycksele Lappmark	Wedin 6873 (UPS)	EU870682	EU870735	LN810880	EU870795
Pycnora sorophora	Sweden	Hermansson 7903a (UPS)	FJ959357	AY853338	AY853387	-
Sarcogyne clavus	U.S.A., New Mexico, Oscura	Kocourkova 10841 (hb. K&K)	OQ171077	OQ184789	OQ195842	-
Sarcogyne nimisii	Italy, Trieste Coast	Nimis 35083 (TSB)	OQ171133	OQ184844	OQ195892	-
Sarcogyne pruinosa	Italia, Campania. Prov. Salerno	Nimis 31664 (TSB)	OQ171125	OQ184836	OQ195885	-
Timdalia intricata	Sweden, Härjedalen	Westberg SAR92 (LD)	LN810866	LN810991	-	LN810755
Trimmatothelopsis oreophila	U.S.A., California, Wildomar (SBBG)	Knudsen 2410 (SBBG)	MK948458	MK948477	-	-
Trimmatothelopsis versipellis	France, Finistère, Plogof	CR25921 (MARSSJ)	KM879336	KM879327	-	-
